# JOSD1-mediated stabilization of SUFU controls pancreatic cancer progression

**DOI:** 10.1038/s41419-026-08954-0

**Published:** 2026-06-05

**Authors:** Kai Li, Jiang Pan, Jujin Zhang, Deyu Kong, Changan Wang, Bin Liang, Lili Han, Biao Liu, Kunpeng Wang, Kexin Gao, Junjie Ji, Yun Zhao, Chao Dong, Liya Ma, Jiayin Peng

**Affiliations:** 1 University of Chinese Academy of Sciences, Beijing, China; 2 School of Life Science, Hangzhou Institute for Advanced Study, University of Chinese Academy of Sciences, Hangzhou, China; 3 School of Life Science and Technology, ShanghaiTech University, Shanghai, China; 4 Department of Medical Oncology, The First Affiliated Hospital of Kunming Medical University, Kunming, China; 5 State Key Laboratory of Molecular Biology, Shanghai Institute of Biochemistry and Cell Biology, Center for Excellence in Molecular Cell Science, Chinese Academy of Sciences, Shanghai, China; 6 Institute for Chinese Medicine Frontier Interdisciplinary Science and Technology, Shaanxi University of Chinese Medicine, Xianyang, China; 7 Bio-research innovation center Suzhou, Suzhou, China

**Keywords:** Cancer, Oncogenes

## Abstract

The Hedgehog (Hh) signaling pathway plays an essential role in embryonic development, and its aberrant activation is associated with pancreatic cancer progression. However, the function of Suppressor of Fused (SUFU), a critical negative regulator of the Hh pathway, in pancreatic cancer has been poorly studied. In this study, we found that *SUFU* knockdown significantly inhibits pancreatic cancer cell growth, migration, and proliferation while inducing apoptosis. We identified a direct interaction between JOSD1 and SUFU, whereby JOSD1 stabilizes SUFU protein by inhibiting its ubiquitination and proteasomal degradation. Public database analyses revealed a positive correlation between *JOSD1* and *SUFU* expression in pancreatic cancer tissues, and immunofluorescence staining of clinical samples further showed that JOSD1 and SUFU are co-localized and expressed at higher levels in pancreatic cancer tissues compared with normal pancreatic tissues. Notably, SUFU regulates pancreatic cancer cell behavior through a non-canonical, Hh-independent mechanism. Depletion of either *JOSD1* or *SUFU* downregulated proliferation-related genes and upregulated apoptosis-related genes, thereby suppressing pancreatic cancer cell growth and promoting apoptosis, with these effects reversed by SUFU restoration. Consistently, silencing of *JOSD1* or *SUFU* significantly attenuates tumor growth and reduces Ki67 expression in vivo, whereas re-expression of SUFU in JOSD1-deficient tumors restores tumor growth and proliferation. Collectively, these findings highlight the functional importance of JOSD1-mediated SUFU stabilization in pancreatic cancer and suggest potential therapeutic strategies targeting this axis.

## Introduction

Pancreatic cancer is a highly aggressive malignancy of the digestive system with a 5-year overall survival (OS) rate of approximately 10%, ranking fourth among gastrointestinal cancers [1, 2]. Its rising incidence and poor prognosis are associated with demographic changes [3]. and complex molecular mechanisms involving KRAS [4]. TGF-β [5]. PI3K/AKT [6]. and Hedgehog (Hh) signaling pathways [7]. which drive tumor progression and therapy resistance. Among these pathways, Hh signaling has emerged as a critical but less thoroughly explored regulator of pancreatic cancer progression.

Aberrant activation of the Hh signaling pathway is a hallmark of pancreatic cancer and has been increasingly recognized as a driver of tumorigenesis and progression [8, 9]. SUFU, a core negative regulator of the Hh signaling pathway, mediates critical functional interactions involving GLI transcription factors [10]. In pancreatic ductal adenocarcinoma cells, SUFU is detected in both the cytoplasm and nucleus, with a positive expression rate of 68.4% [11]. Additionally, a novel splice variant of *SUFU* mRNA has been identified in pancreatic cancer, which may affect protein function through structural remodeling and potentially act as an oncogenic factor [12]. Beyond its canonical role in regulating Hh signaling, SUFU can influence tumor progression through Hh-independent mechanisms across different cancer types, including pancreatic cancer. SUFU has been implicated in regulating ferroptosis sensitivity and tumor cell migration through Hh-independent pathways across different cancer types, and can also promote GLI activity independently of upstream Hh signaling in pancreatic cancer [13–15]. These findings suggest that SUFU may regulate tumor cell function through both Hh-dependent and Hh-independent mechanisms, highlighting its complex and context-dependent role in cancer progression and warranting further investigation into its regulatory mechanisms.

SUFU protein stability is dynamically regulated by the ubiquitin-proteasome system [16, 17], raising the possibility that deubiquitination may represent an additional regulatory layer controlling SUFU protein stability. Deubiquitinating enzymes (DUBs) counteract ubiquitin-dependent degradation to preserve protein stability. Dysfunction of this process has emerged as a central mechanism driving tumorigenesis across multiple cancer types [18, 19]. The Machado-Joseph disease (MJD) family represents the smallest class of DUB enzymes and is classically associated with the neurodegenerative disease Machado-Joseph disease [20]. This family comprises four cysteine protease members in humans: Ataxin-3, Ataxin-3-like protein (ATXN3L), Josephin-1 (JOSD1), and Josephin-2 (JOSD2) [21]. Beyond their roles in neurodegeneration [22–25]. MJD family proteins have been increasingly implicated in cancer biology, where they promote breast cancer cell proliferation [26] and participate in the regulation of key signaling pathways, including PTEN/PI3K-AKT and p53 signaling, as well as DNA damage repair [27–30]. Notably, MJD family proteins exhibit diverse and multifaceted functions in tumorigenesis. Clinical observations further indicate a reduced cancer mortality rate among MJD patients compared to the general population [31]. suggesting a protective effect potentially associated with altered ubiquitin dynamics. Among these, JOSD1 has emerged as an important regulator of tumor progression. JOSD1 enhances inhibition of interferon type I (IFN-I) signaling by deubiquitinating SOCS1 [32] and induces chemoresistance in ovarian cancer by stabilization of MCL1 [33]. These findings highlight the important role of MJD family deubiquitinating enzymes in regulating protein stability and tumor progression. Given that the stability of SUFU is tightly controlled by the ubiquitin-proteasome system, it is conceivable that MJD family DUBs may also participate in regulating SUFU stability. Accordingly, elucidating the specific mechanisms by which MJD family DUBs regulate SUFU stability may uncover novel therapeutic targets for pancreatic cancer.

In this study, we elucidate the interplay between the MJD family and SUFU, identifying JOSD1 as a deubiquitinating enzyme that directly interacts with SUFU. We perform biochemical assays to characterize the regulatory effects of JOSD1 on SUFU stability and function. We further evaluate how JOSD1-mediated modulation of SUFU influences pancreatic cancer cell behavior in vitro using assays of proliferation, migration, and invasion. Finally, we investigate the in vivo effects of JOSD1 on pancreatic tumor growth and progression via SUFU regulation using xenograft models. Our findings establish a JOSD1-SUFU regulatory axis with potential relevance to pancreatic cancer pathogenesis.

## Materials and methods

### Patient specimens and ethics statement

PDAC tissue samples were collected at Changhai Hospital and processed into paraffin-embedded tissue sections, which were kindly provided by Prof. Dong Gao [34]. All patients provided written informed consent for the use of their clinical data and surgical specimens, as well as for the publication of potentially identifying clinical information. The study was conducted in accordance with national guidelines and was approved by the Ethics Committee of Changhai Hospital (Approval No. CHEC2018-111). In addition to local IRB approval, this study was reviewed and approved by the Chinese Ministry of Science and Technology (MOST) for compliance with human genetic resource regulations (Approval No. 2021BAT1264).

### Total RNA extraction, RT-PCR, and RT-qPCR

For RNA extraction from cells, using a 6 cm dish as an example. Cells were lysed with 1 mL of Trizol reagent. After centrifugation, the supernatant was collected, and 1/5 volume of chloroform was added. The mixture was vortexed and then incubated at room temperature for 5 min before centrifugation. The supernatant was collected again and an equal volume of isopropanol was added. The mixture was incubated at −20 °C for 1–2 h and then centrifuged. The supernatant was discarded, and the pellet was washed twice with 75% ethanol. RNA was dissolved in DEPC water, and the RNA concentration was measured. The extracted RNA was reverse transcribed into cDNA using the HiScript IV RT SuperMix for qPCR kit, followed by quantitative PCR detection using the AceQ Universal SYBR qPCR Master Mix kit (The RT-qPCR primers are listed in Table 1).

**Table 1:** List of RT-qPCR primer sequences used in this study.

Gene name	Forward oligo	Reverse oligo
*GAPDH*	TGCACCACCAACTGCTTAG	GGATGCAGGGATGATGTTC
*JOSD1*	TGGGGTCCACTGAAACTGC	CTCGCCTCCAATCCACTCG
*SUFU*	CACGCCATCTACGGAGAGTG	GTACTTGACGATAGCGGTAACC
*TNF*	GAGGCCAAGCCCTGGTATG	CGGGCCGATTGATCTCAGC
*IL32*	TGGCGGCTTATTATGAGGAGC	CTCGGCACCGTAATCCATCTC
*SORBS2*	ACAACCCACCCTACAGTGCT	GGACGCATCCTTAAAGGCATTG
*CLIC3*	CCTCAAGGGCGTACCTTTCAC	GTCGCTGTCATAGAGCAGGA
*MMP1*	GGGGCTTTGATGTACCCTAGC	TGTCACACGCTTTTGGGGTTT
*ANXA10*	GCTGGCCTCATGTACCCAC	CAAGCAGTAGGCTTCTCGC
*CEACAM6*	TCAATGGGACGTTCCAGCAAT	CACTCCAATCGTGATGCCGA
*ETV1*	GGCCCCAGGCAGTTTTATGAT	GATCCTCGCCGTTGGTATGT
*PIWIL1*	TCAGGAGTTATCGTTAGCAGAGA	TGTCAGCCGGAAATGGTTAGT
*F5*	GAACCATCATAAGGTCTCAGCC	CCTCTGCTCACGAGTTATTTTCT
*SLC14A1*	TGGCTGGGAACAGTGGTCT	AGTCTCCCTTGTCCGAAAAGA
*GLI1*	GACATACCCCACCTCCCTCT	ACTGCAGCTCCCCCAATTTT
*GLI2*	CAAGGCACCGCATCTGTGAT	CCAGGATCCCACTTTTGGCT
*PTCH1*	CCACAGAAGCGCTCCTACA	CTGTAATTTCGCCCCTTCC

### Cell culture

BxPC-3 was maintained in RPMI 1640 + 10% FBS and 1% penicillin–streptomycin, PANC-1 and HEK293T cell lines were maintained in DMEM + 10% FBS and 1% penicillin–streptomycin. All cells were cultured at 37 °C in a humidified incubator with 5% CO₂. All cells were derived from the CAS Cell Bank. Cells were passaged every 2–3 days to maintain exponential growth. Mycoplasma contamination was excluded by PCR using published primer sets, and only mycoplasma-free cells were used for experiments to ensure data integrity.

### Plasmid construction and cell transfection

Using pcDNA3.1 as the vector, Myc-SUFU and Flag-MJD family plasmids were constructed. sh-SUFU, sh-JOSD1, oe-GLI1 and oe-SUFU plasmids were constructed using pLKO.1 as the vector (The sequences of siRNA and shRNA are listed in Tables 2 and 3). Successful plasmid construction was achieved through transformation, identification, cultivation, and extraction of the plasmids. When the cell condition was good under the microscope and the cell density reached around 70%, Lipofectamine 3000 was used for cell transfection. The cells were gently agitated and then placed in the incubator for culture.

**Table 2:** List of si-RNA sequences used in this study.

Gene name	Forward oligo	Reverse oligo
Negative control	UUCUCCGAACGUGUCACGUTT	ACGUGACACGUUCGGAGAATT
*JOSD1*	CUAACGUCAUGGGCUUCAUTT	AUGAAGCCCAUGACGUUAGTT
*SUFU*	GACCCUGGUUACAAAUUCUTT	AGAAUUUGUAACCAGGGUCTT

**Table 3:** List of sh-RNA sequences used in this study.

Gene name	Forward oligo	Reverse oligo
Negative control	CCGGTTCTCCGAACGTGTCACGTTTCTCGAGAAACGTGACACGTTCGGAGAATTTTTG	AATTCAAAAATTCTCCGAACGTGTCACGTTTCTCGAGAAACGTGACACGTTCGGAGAA
*JOSD1*	CCGGCTAACGTCATGGGCTTCATTTCTCGAGAAATGAAGCCCATGACGTTAGTTTTTG	AATTCAAAAACTAACGTCATGGGCTTCATTTCTCGAGAAATGAAGCCCATGACGTTAG
*SUFU*	CCGGGACCCTGGTTACAAATTCTTTCTCGAGAAAGAATTTGTAACCAGGGTCTTTTTG	AATTCAAAAAGACCCTGGTTACAAATTCTTTCTCGAGAAAGAATTTGTAACCAGGGTC

### Lentivirus production and stable cell lines

By co-transfecting HEK293T cells with PEI and pMD2.G, psPAX2 and the target plasmid in a ratio of 3.5:7:15, lentiviral particles were prepared. The virus supernatant was collected 48 h and 72 h after transfection, filtered through a 0.45 μm membrane, concentrated using PEG8000, and re-suspended in Opti-MEM. The target cells were infected with 50 μL of the virus suspension + 8 μg/mL polybrene. Stable knockdown and overexpression cell lines were established by antibiotic selection.

### Co-immunoprecipitation

Cells were lysed using NP-40 lysis buffer containing protease inhibitors, and the supernatant was collected after centrifugation. A small amount of the lysate was reserved as whole-cell lysate for western blot analysis. The remaining lysate was incubated with the appropriate antibodies at 4 °C overnight, followed by the addition of an appropriate amount of agarose beads and incubation at 4 °C for 2 h. After centrifugation, the supernatant was discarded, and the pellet was washed four times with NP-40 lysis buffer. The precipitated complexes were re-suspended in an appropriate amount of SDS loading buffer and used for western blot analysis.

### Western blot

Prepare an SDS-PAGE gel with an appropriate concentration based on the protein size. After the gel has solidified, load an appropriate amount of protein samples and perform electrophoresis. Once the electrophoresis is completed, transfer the proteins to a PVDF membrane. Block the membrane with 5% BSA for 1 h. Incubate with the primary antibody at 4 °C overnight. The next day, wash the membrane three times with TBS-T, add the HRP-conjugated secondary antibody and incubate at room temperature for 1 h, then wash the membrane again (The primary and secondary antibodies are listed in Table 4). Finally, visualize the results using an imaging system.

**Table 4:** List of experimental antibodies used in this study.

Antibody	Vendor	Catalog number
SUFU (C81H7) Rabbit mAb	CST	2522S
GLI1 (C68H3) Rabbit mAb	CST	3538S
GLI2 Rabbit pAb	ABclonal	A16864
Ubiquitin (P4D1) Mouse mAb	CST	3936S
Normal Rabbit IgG	CST	2729S
Anti-JOSD1 antibody	Abcam	ab118221
Rabbit Anti-Ki67 antibody	Abcam	ab15580
Mouse Anti-Flag-tag mAb	Sigma-Aldrich	F3165-5 mg
Rabbit Anti-Myc-tag pAb	MBL	562-5
Mouse Anti-Myc-tag mAb	ABclonal	AE010_100 μL
Flag-tag Rabbit mAb	ABclonal	AE063_100 μL
Goat & Rabbit IgG (H + L) HRP	Thermo Fisher Invitrogen	G21234
Goat & Mouse IgG (H + L) HRP	Thermo Fisher Invitrogen	G21040

### Immunofluorescence staining

Paraffin-embedded tissue sections were processed using a tyramide signal amplification (TSA)-based multiplex immunofluorescence staining system (Akoya Biosciences). Briefly, sections were baked at 60 °C for 1.5 h, followed by deparaffinization, rehydration, and antigen retrieval. After cooling to room temperature, tissue sections were outlined and blocked with 2% BSA in TBS-T for 20 min. Sections were then incubated with primary antibodies at 4 °C overnight (The primary antibodies are listed in Table 4). The next day, sections were washed with TBS-T and incubated with HRP-conjugated secondary antibodies (Opal polymer HRP Ms + Rb) at room temperature for 1 h. After washing, Opal fluorophores were applied and incubated for 10 min in the dark. For multiplex staining, antigen retrieval was repeated to strip bound antibodies before incubation with subsequent primary antibodies, followed by detection using spectrally distinct Opal fluorophores. After completing all staining cycles, nuclei were counterstained with DAPI for 5 min. Sections were washed and mounted with an aqueous mounting medium.

### Cell function experiments

Cell growth was detected using the direct cell counting and CCK-8 assays. For CCK-8 assay, 10 µL of CCK-8 solution was added to a 96-well plate and incubated for 2 h. The OD value at a wavelength of 450 nm was measured using a microplate reader to calculate the relative cell viability and plot the growth curve.

Cell apoptosis was detected using the Annexin V-FITC/PI double staining method. The cell suspension was mixed with Annexin V-FITC staining solution and PI staining solution, vortexed, and then incubated in the dark at room temperature. Flow cytometry was used for detection, and the proportion of each cell population was calculated using analysis software.

The ability to form colonies was detected using the colony formation assay. After colony formation, the colonies were fixed with 4% PFA, stained with crystal violet, and photographed for record. The colonies were then dissolved in 10% acetic acid, and the OD value at 590 nm was measured using a microplate reader.

Cell migration and wound healing were studied using the scratch assay. When the cell density reached 90–100%, a linear scratch was made with a scratch tool. Photos were taken at 0 h, 8 h, and 16 h. The change in scratch width was measured using image analysis software to calculate the migration rate or closure rate.

### Subcutaneous tumor formation in nude mice

The nude mice used in the experiment were 4–6-week-old BALB/c nude mice, housed five per cage, and had been acclimated for 1 week before the experiment. During the experiment, the mice were anesthetized, and a cell suspension was drawn up using an insulin syringe to inject ~200 µL subcutaneously into the back or flank. The inoculation time was recorded, and the recovery of the mice was observed. Starting from day 7, the length and width of the tumor were measured every 2–3 days to calculate the volume using the formula *V* = (*L* × *W*^2^)/2, and a growth curve was plotted. When the tumor volume reached 1000 mm^3^ or at the end of the experiment, the mice were euthanized, and the tumor tissue was weighed, photographed, and processed further. Mice were housed under specific pathogen-free (SPF) conditions. All animal studies were reviewed and approved by the Institutional Animal Care and Research Advisory Committee of the Shanghai Institute of Biochemistry and Cell Biology in accordance with institutional guidelines.

### RNA-seq library preparation, sequencing and bioinformatics analysis

Extract total RNA using TRIzol reagent. Assess RNA quality through NanoDrop (OD_260/280_ = 1.8–2.2) and Agilent 2100 bioanalyzer (RIN ≥ 8.0). Prepare a strand-specific library using the MGIEasy RNA Library Preparation Kit (MGI) and sequenced on DNBSEQ-G400 (PE150). Filter the raw data using SOAPnuke v2.1.0 and align the clean reads to GRCh38 using HISAT2. Quantify gene expression (FPKM) using RSEM v1.3.3 through Bowtie2 and generate raw counts using featureCounts (Subread). Conduct independent differential expression analysis and define genes with |log2FC | >0.5 and *P.*adj < 0.05 as significantly differentially expressed genes. Perform GO and KEGG enrichment analysis using clusterProfiler (*P*.adjust <0.05). All operations follow the standard procedures of Wuhan Frazang Bioinformatics.

### Statistical analysis

All statistical analyses in this study were based on the results of biological and technical replicates of the experimental data (*n* ≥ 3). Depending on the different experimental groupings, appropriate statistical analysis methods were selected: unpaired Student’s *t* test for comparisons between two independent groups, and one-way ANOVA or two-way ANOVA for multiple group comparisons. Statistical significance is indicated by symbols: *P < 0.05, **P < 0.01, ***P < 0.001, ****P < 0.0001, and ns indicates no statistical significance. All data are presented as mean ± SEM, except for the analysis of the expression levels of *SUFU* and *JOSD1* in the TCGA and GTEx databases, where mean ± SD was used.

## Results

### SUFU is essential for pancreatic cancer cell proliferation

**Fig. 1:**
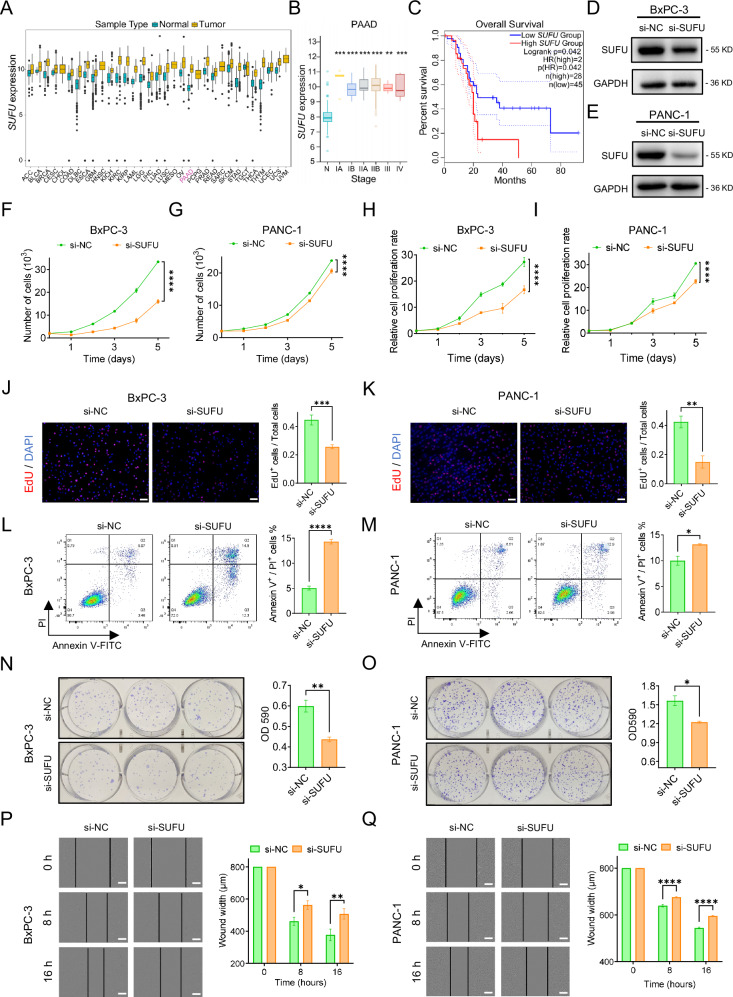
SUFU is essential for pancreatic cancer cell proliferation. **A** Pan-cancer expression profile of SUFU based on TCGA data. **B** Expression of *SUFU* in different stages of pancreatic cancer (N: Normal (*n* = 171), IA: Stage IA (*n* = 5), IB: Stage IB (*n* = 15), IIA: Stage IIA (*n* = 28), IIB: Stage IIB (*n* = 119), III: Stage III (*n* = 3), IV: Stage IV (*n* = 5). Median ± IQR. **P < 0.01, ***P < 0.001. Wilcoxon rank-sum test). **C** Kaplan–Meier analysis of overall survival stratified by SUFU expression in pancreatic cancer. **D** Western blot analysis of SUFU knockdown efficiency in BxPC-3 cells following siRNA transfection. **E** Western blot analysis of SUFU knockdown efficiency in PANC-1 cells following siRNA transfection. **F** Cell proliferation of BxPC-3 cells following SUFU knockdown was assessed by cell counting (*n* = 3; mean ± SEM; ****P < 0.0001; two-way ANOVA). **G** Cell proliferation of PANC-1 cells following SUFU knockdown was assessed by cell counting (*n* = 3; mean ± SEM; ****P < 0.0001; two-way ANOVA). **H** Relative cell proliferation of BxPC-3 cells following SUFU knockdown was assessed by CCK-8 assay (*n* = 4; Mean ± SEM; ****P < 0.0001; two-way ANOVA). **I** Relative cell proliferation of PANC-1 cells following SUFU knockdown was assessed by CCK-8 assay (*n* = 4. Mean ± SEM. ****P < 0.0001. Two-way ANOVA). **J** EdU staining showing the proportion of proliferating BxPC-3 cells following SUFU knockdown (bar, 50 µm. *n* = 3. Mean ± SEM. ***P < 0.001. Unpaired Student’s *t* test). **K** EdU staining showing the proportion of proliferating PANC-1 cells following SUFU knockdown (bar, 50 µm. *n* = 3. Mean ± SEM. **P < 0.01. Unpaired Student’s *t* test). **L** Annexin V/PI staining showing the proportion of apoptotic BxPC-3 cells following SUFU knockdown (*n* = 4. Mean ± SEM. ****P < 0.0001. Unpaired Student’s *t* test). **M** Annexin V/PI staining showing the proportion of apoptotic PANC-1 cells following SUFU knockdown (*n* = 4. Mean ± SEM. *P < 0.05. Unpaired Student’s *t* test). **N** Colony formation assay showing the clonogenic ability of BxPC-3 cells following SUFU knockdown (*n* = 3. Mean ± SEM. **P < 0.01. Unpaired Student’s *t* test). **O** Colony formation assay showing the clonogenic ability of PANC-1 cells following SUFU knockdown (*n* = 3. Mean ± SEM. *P < 0.05. Unpaired Student’s *t* test). **P** Scratch assay showing the migration of BxPC-3 cells following SUFU knockdown (bar, 200 µm. *n* = 4. Mean ± SEM. *P < 0.05, **P < 0.01. Two-way ANOVA). **Q** Scratch assay showing the migration of PANC-1 cells following SUFU knockdown (bar, 200 µm. *n* = 6. Mean ± SEM. ****P < 0.0001. Two-way ANOVA).

To investigate the functional role of SUFU in pancreatic cancer, we first performed a pan-cancer analysis of SUFU expression using data from the TCGA (The Cancer Genome Atlas) and GTEx (Genotype-Tissue Expression) databases. We found that *SUFU* expression is upregulated across multiple cancer types, with particularly pronounced elevation in pancreatic cancer, where it is significantly higher in tumor tissues than in normal controls (Fig. 1A). Consistently, *SUFU* levels remained higher across various clinical stages of pancreatic cancer compared to normal tissues (Fig. 1B). To further assess its prognostic relevance, we analyzed patient survival using the GEPIA (Gene Expression Profiling Interactive Analysis) database. Patients with high *SUFU* expression exhibited significantly shorter overall survival than those with low expression (Fig. 1C), indicating an inverse correlation between *SUFU* levels and patient prognosis.

We next examined the role of SUFU in pancreatic cancer cells using two representative cell lines, BxPC-3 and PANC-1. Small interfering RNA (siRNA) was employed to silence *SUFU* in both cell lines, and Western blotting was performed to assess the protein levels of SUFU and confirm knockdown efficiency. siRNA-mediated *SUFU* knockdown significantly reduced endogenous SUFU protein levels in both BxPC-3 and PANC-1 cells (Fig. 1D, E), validating the effectiveness of the siRNA strategy. To determine whether SUFU affects pancreatic cancer cell proliferation, we next assessed cell growth following SUFU silencing using direct cell counting and CCK-8 assays. Both assays consistently showed that SUFU depletion significantly suppressed cell proliferation in BxPC-3 and PANC-1 cells (Fig. 1F–I). EdU (5-ethynyl-2’-deoxyuridine) incorporation assays further confirmed this effect, showing a marked reduction in the proportion of proliferating cells in BxPC-3 cells following SUFU knockdown (Fig. 1J), with similar results observed in PANC-1 cells (Fig. 1K). To further characterize the effects of SUFU depletion, we assessed changes in cell apoptosis, colony formation, and migration in BxPC-3 and PANC-1 cells. Annexin V/PI staining revealed a significant increase in apoptotic cells upon SUFU knockdown in both cell lines (Fig. 1L, M). Colony formation assays showed that SUFU depletion markedly reduced clonogenic capacity in both BxPC-3 and PANC-1 cells (Fig. 1N, O). Consistently, scratch assays demonstrated that SUFU knockdown significantly impaired cell migration in both cell lines (Fig. 1P, Q). Collectively, these results indicate that SUFU promotes pancreatic cancer cell proliferation, survival, and migratory capacity, and is associated with disease progression and poor patient prognosis.

### JOSD1 stabilizes SUFU by inhibiting ubiquitination and proteasomal degradation

**Fig. 2:**
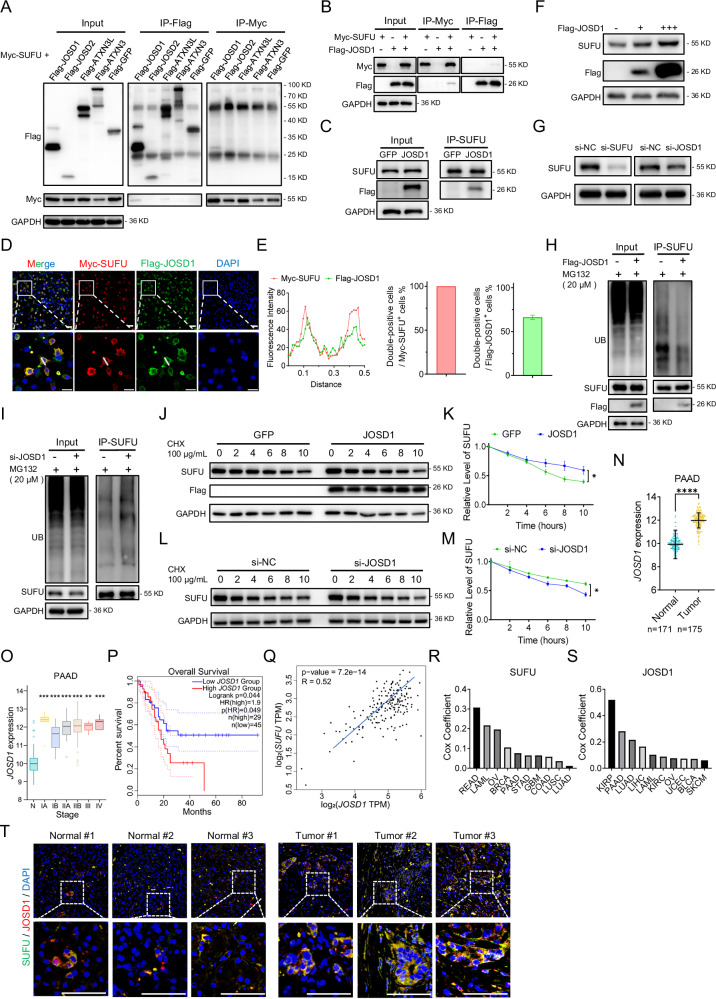
JOSD1 stabilizes SUFU by inhibiting its ubiquitination and is positively associated with SUFU in pancreatic cancer. **A** JOSD1 was identified among MJD family members as a SUFU-interacting protein by co-immunoprecipitation (Co-IP). **B** The interaction between JOSD1 and SUFU was verified by exogenous Co-IP. **C** The interaction between JOSD1 and endogenous SUFU was validated by semi-endogenous Co-IP. **D** Immunofluorescence staining of Myc-SUFU and Flag-JOSD1 (bar, 50 µm). **E** Fluorescence intensity profiles of Myc-SUFU and Flag-JOSD1 show overlapping distributions; quantification of double-positive cells relative to single-positive cells. **F** Endogenous SUFU protein levels increased in a dose-dependent manner upon JOSD1 overexpression. **G** Endogenous SUFU protein levels decreased following JOSD1 knockdown. **H** Overexpression of JOSD1 decreased the ubiquitination level of SUFU. **I** Knockdown of JOSD1 increased the ubiquitination level of SUFU. **J** Western blot analysis of SUFU protein levels following cycloheximide (CHX) treatment at the indicated time points under JOSD1 overexpression. **K** Quantification of SUFU protein levels in (**J**) (*n* = 4. Mean ± SEM. *P < 0.05. Two-way ANOVA). **L** Western blot analysis of SUFU protein levels following CHX treatment at the indicated time points after JOSD1 knockdown. **M** Quantification of SUFU protein levels in (**L**) (*n* = 3. Mean ± SEM. *P < 0.05. Two-way ANOVA). **N** Expression of JOSD1 in pancreatic cancer based on public datasets. (Normal (*n* = 171), Tumor (*n* = 175). Mean ± SD. ****P < 0.0001. Unpaired Student’s *t* test). **O** Expression of JOSD1 across different stages of pancreatic cancer. N: normal (*n* = 171); IA: stage IA (*n* = 5); IB: stage IB (*n* = 15); IIA: stage IIA (*n* = 28); IIB: stage IIB (*n* = 119); III: stage III (*n* = 3); IV: stage IV (*n* = 5) (median ± IQR. **P < 0.01, ***P < 0.001. Wilcoxon rank-sum test). **P** Kaplan–Meier analysis of overall survival stratified by JOSD1 expression in pancreatic cancer. **Q** Positive correlation between JOSD1 and SUFU expression in pancreatic cancer. **R** Association between SUFU expression and cancer risk across multiple tumor types. **S** Association between JOSD1 expression and cancer risk across multiple tumor types. **T** Immunofluorescence staining of SUFU and JOSD1 in normal pancreatic tissues and pancreatic cancer tissues (bar, 50 µm).

Given the functional role of SUFU in pancreatic cancer, we next sought to investigate the regulatory mechanisms underlying its expression. As SUFU protein stability is dynamically regulated by the ubiquitin-proteasome system, and members of the MJD deubiquitinating enzyme family are known to modulate protein stability and play important roles in tumor progression, we investigated whether MJD family proteins regulate SUFU. To identify potential interactions between MJD family deubiquitinating enzymes and SUFU, we constructed Myc-SUFU and Flag-MJD family expression plasmids and performed co-immunoprecipitation (Co-IP) assays in HEK293T cells. Cells were co-transfected with pcDNA3.1-Myc-SUFU and various pcDNA3.1-Flag-MJD constructs, followed by Co-IP analysis 24 h post-transfection. These experiments revealed a specific interaction between JOSD1 and SUFU (Fig. 2A). To validate this interaction, we co-transfected pcDNA3.1-Myc-SUFU and pcDNA3.1-Flag-JOSD1 into HEK293T cells, and Co-IP analysis reaffirmed the interaction between SUFU and JOSD1 (Fig. 2B). Furthermore, semi-endogenous Co-IP assays following transfection of pcDNA3.1-Flag-JOSD1 showed that JOSD1 interacts with endogenous SUFU (Fig. 2C). To assess their intracellular localization, we co-transfected pcDNA3.1-Myc-SUFU and pcDNA3.1-Flag-JOSD1 into HEK293T cells and performed immunofluorescence staining. JOSD1 and SUFU were found to co-localize in the cytoplasm (Fig. 2D), which was further supported by quantitative analysis of fluorescence intensity and the proportion of co-localized cells (Fig. 2E). Notably, SUFU expression was largely restricted to JOSD1-positive cells, whereas JOSD1 was also detected in cells lacking SUFU. This asymmetric distribution is consistent with a role for JOSD1 in maintaining SUFU stability.

Given this interaction, we next explored whether JOSD1 modulates SUFU protein levels. HEK293T cells were transfected with increasing amounts of pcDNA3.1-Flag-JOSD1, and SUFU protein levels were analyzed by Western blotting. JOSD1 overexpression led to a dose-dependent increase in endogenous SUFU protein levels (Fig. 2F). Conversely, siRNA-mediated depletion of JOSD1 resulted in a marked reduction in SUFU protein levels, as assessed by Western blotting 48 h post-transfection (Fig. 2G). To determine whether JOSD1 regulates SUFU ubiquitination, HEK293T cells were transfected with pcDNA3.1-Flag-JOSD1 and treated with the proteasome inhibitor MG132. Co-IP analysis showed that JOSD1 overexpression markedly reduced the ubiquitination of SUFU (Fig. 2H). In contrast, JOSD1 depletion led to increased ubiquitination of endogenous SUFU following MG132 treatment (Fig. 2I), indicating that JOSD1 stabilizes SUFU by inhibiting its ubiquitination.

Cycloheximide (CHX), a eukaryotic protein synthesis inhibitor, binds to the 60S ribosomal subunit and blocks translation elongation by preventing eEF2-mediated tRNA translocation. This property makes CHX a widely used tool for studying protein stability. To further evaluate the effect of JOSD1 on SUFU protein stability, we performed CHX chase assays. HEK293T cells were transfected with pcDNA3.1-Flag-JOSD1 and treated with CHX 24 h post-transfection, followed by collection of protein lysates at the indicated time points. Western blot analysis revealed that SUFU protein levels were maintained at higher levels in JOSD1-overexpressing cells compared to controls (Fig. 2J). Quantitative analysis revealed that JOSD1 overexpression significantly extended the half-life of SUFU (Fig. 2K). Conversely, knockdown of *JOSD1* via siRNA accelerated SUFU degradation, as evidenced by reduced SUFU levels over time (Fig. 2L) and a shortened protein half-life (Fig. 2M). Collectively, these findings demonstrate that JOSD1 stabilizes SUFU by inhibiting its ubiquitination and proteasomal degradation.

### JOSD1 is upregulated in pancreatic cancer and correlates with SUFU expression and poor prognosis

Based on the regulatory role of JOSD1 in controlling SUFU stability, we next examined whether JOSD1 is dysregulated in pancreatic cancer. Analysis of TCGA and GTEx datasets showed that *JOSD1* expression was significantly elevated in tumors tissues compared to normal controls (Fig. 2N). *JOSD1* expression remained higher across different clinical stages of pancreatic cancer (Fig. 2O). Using the GEPIA platform, we further assessed the prognostic relevance of JOSD1 and found that high JOSD1 expression was associated with reduced overall survival (Fig. 2P), mirroring the pattern observed for *SUFU*. Correlation analysis showed a significant positive correlation between *JOSD1* and *SUFU* expression in pancreatic cancer samples (Fig. 2Q). To extend these findings, we employed OncoLnc to calculate Cox proportional hazards coefficients for *SUFU* and *JOSD1* across various tumor types. Notably, pancreatic cancer ranked among the top tumor types showing strong prognostic associations for both genes (Fig. 2R, S), indicating that elevated expression of *SUFU* and *JOSD1* is linked to poor patient outcomes in pancreatic cancer. To further validate these findings, we performed immunofluorescence staining on pancreatic cancer tissue sections. SUFU and JOSD1 were found to co-localize and exhibit elevated expression in tumor tissues compared to normal controls (Fig. 2T), supporting their clinical relevance.

### JOSD1 modulates pancreatic cancer cell functions in vitro

**Fig. 3:**
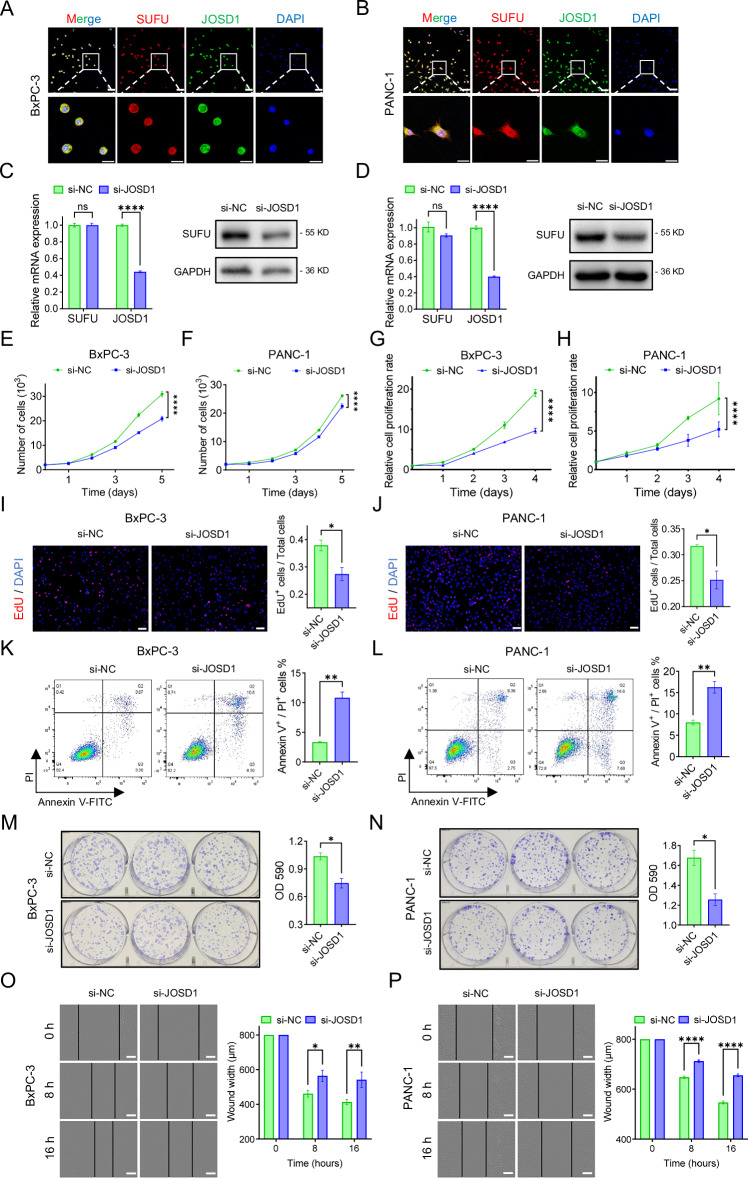
JOSD1 modulates pancreatic cancer cells proliferation in vitro. **A** Immunofluorescence staining of endogenous SUFU and JOSD1 in BxPC-3 cells (bar, 50 µm). **B** Immunofluorescence staining of endogenous SUFU and JOSD1 in PANC-1 cells (bar, 50 µm). **C** mRNA levels of SUFU and JOSD1 in BxPC-3 cells were analyzed by RT-qPCR; SUFU protein levels following JOSD1 knockdown were assessed by western blot (*n* = 5. Mean ± SEM. ****P < 0.0001. ns no significance. Two-way ANOVA). **D** mRNA levels of SUFU and JOSD1 in PANC-1 cells were analyzed by RT-qPCR; SUFU protein levels following JOSD1 knockdown were assessed by western blot (*n* = 5. Mean ± SEM. ****P < 0.0001. ns, no significance. Two-way ANOVA). **E** Cell proliferation of BxPC-3 cells following JOSD1 knockdown was assessed by cell counting. (*n* = 3. Mean ± SEM. ****P < 0.0001. Two-way ANOVA). **F** Cell proliferation of PANC-1 cells following JOSD1 knockdown was assessed by cell counting (*n* = 4. Mean ± SEM. ****P < 0.0001. Two-way ANOVA). **G** Relative cell proliferation of BxPC-3 cells following JOSD1 knockdown was assessed by CCK-8 assay (*n* = 5. Mean ± SEM. ****P < 0.0001. Two-way ANOVA). **H** Relative cell proliferation of PANC-1 cells following JOSD1 knockdown was assessed by CCK-8 assay (*n* = 5. Mean ± SEM. ****P < 0.0001. Two-way ANOVA). **I** EdU staining showing the proportion of proliferating BxPC-3 cells following JOSD1 knockdown (bar, 50 µm. *n* = 3. Mean ± SEM. *P < 0.05. Unpaired Student’s *t* test). **J** EdU staining showing the proportion of proliferating PANC-1 cells following JOSD1 knockdown (bar, 50 µm. *n* = 3. Mean ± SEM. *P < 0.05. Unpaired Student’s *t* test). **K** Annexin V/PI staining showing the proportion of apoptotic BxPC-3 cells following JOSD1 knockdown (*n* = 3. Mean ± SEM. **P < 0.01. Unpaired Student’s *t* test). **L** Annexin V/PI staining showing the proportion of apoptotic PANC-1 cells following JOSD1 knockdown (*n* = 3. Mean ± SEM. **P < 0.01. Unpaired Student’s *t* test). **M** Colony formation assay showing the clonogenic ability of BxPC-3 cells following JOSD1 knockdown (*n* = 3. Mean ± SEM. *P < 0.05. Unpaired Student’s *t* test). **N** Colony formation assay showing the clonogenic ability of PANC-1 cells following JOSD1 knockdown (*n* = 3. Mean ± SEM. *P < 0.05. Unpaired Student’s *t* test). **O** Scratch assay showing the migration of BxPC-3 cells following JOSD1 knockdown (bar, 200 µm. *n* = 4. Mean ± SEM. *P < 0.05, **P < 0.01. Two-way ANOVA). **P** Scratch assay showing the migration of PANC-1 cells following JOSD1 knockdown (bar, 200 µm. *n* = 6. Mean ± SEM. ****P < 0.0001. Two-way ANOVA).

To investigate the functional role of JOSD1 in pancreatic cancer cells, we first examined its subcellular localization together with SUFU. IF staining revealed clear co-localization of endogenous JOSD1 and SUFU in both BxPC-3 (Fig. 3A) and PANC-1 cells (Fig. 3B). To assess whether JOSD1 regulates SUFU expression, we silenced *JOSD1* using siRNA in BxPC-3 and PANC-1 cells. RNA and protein samples were harvested 24 h post-transfection. RT-qPCR analysis showed efficient JOSD1 knockdown, while *SUFU* mRNA levels remained unchanged (Fig. 3C, D). In contrast, Western blot analysis revealed that *JOSD1* knockdown significantly reduced endogenous SUFU protein levels in both cell lines (Fig. 3C, D). These findings suggest that JOSD1 regulates SUFU at a post-transcriptional level by modulating its protein stability rather than its mRNA expression.

To evaluate the role of JOSD1 on pancreatic cancer cells, we silenced *JOSD1* using siRNA and evaluated cell proliferation through direct cell counting and the CCK-8 assays. Both assays consistently showed that JOSD1 depletion significantly suppressed proliferation in pancreatic cancer cells (BxPC-3 and PANC-1) compared with controls (Fig. 3E–H). EdU incorporation assays further showed reduced proliferative capacity following JOSD1 silencing (Fig. 3I, J). We next assessed the effects of *JOSD1* knockdown on apoptosis, colony formation, and migration. Annexin V/PI staining indicated a significant increase in apoptotic cells (Fig. 3K, L). Colony formation assays demonstrated a marked reduction in clonogenic capacity (Fig. 3M, N), and scratch assays revealed impaired migration (Fig. 3O, P). Collectively, these results demonstrate that JOSD1 promotes pancreatic cancer cell proliferation, survival, and migration.

### JOSD1-SUFU regulates pancreatic cancer cell growth through an Hh-independent transcriptional program

**Fig. 4:**
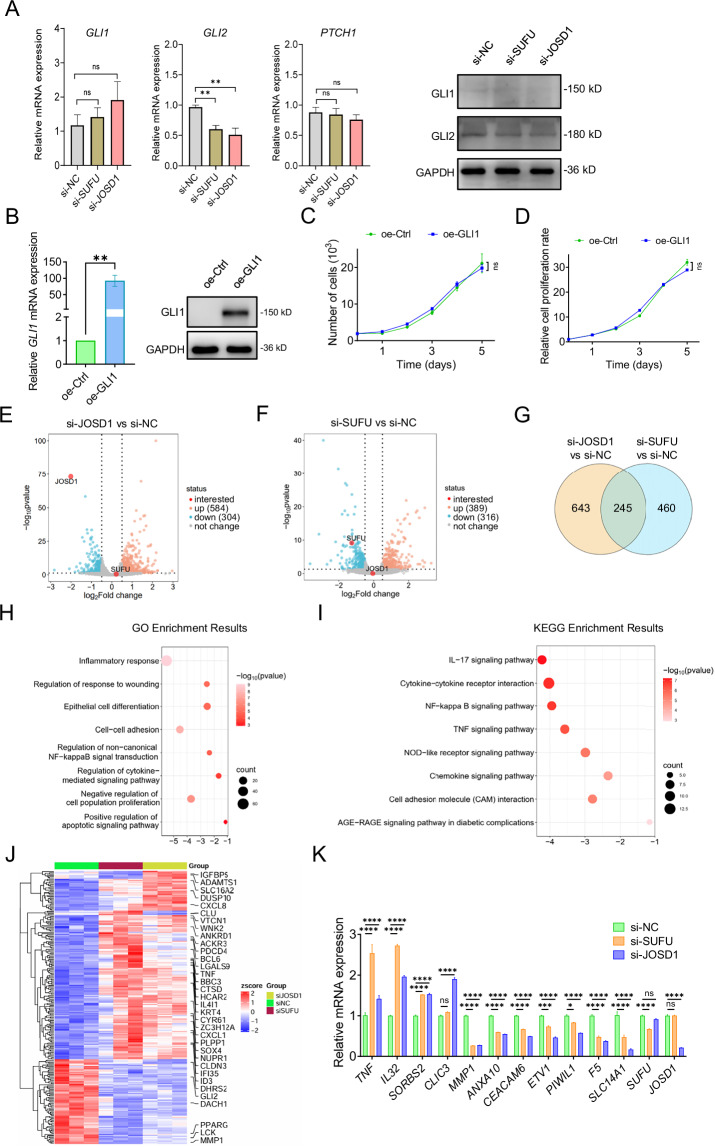
RNA-seq analysis of downstream gene changes following JOSD1 and SUFU knockdown. **A** mRNA levels of GLI1, GLI2, and PTCH1 in BxPC-3 cells following JOSD1 or SUFU knockdown were analyzed by RT-qPCR; protein levels of GLI1 and GLI2 were assessed by western blot (*n* = 3. Mean ± SEM. **P < 0.01. ns no significance. One-way ANOVA). **B** Overexpression efficiency of GLI1 in BxPC-3 cells following viral transduction was confirmed by RT-qPCR and western blot (*n* = 3. Mean ± SEM. **P < 0.01. Unpaired Student’s *t* test). **C** Cell proliferation of BxPC-3 cells following GLI1 overexpression was assessed by cell counting (*n* = 3. Mean ± SEM. ns, no significance. Two-way ANOVA). **D** Relative cell proliferation of BxPC-3 cells following GLI1 overexpression was assessed by CCK-8 assay (*n* = 3. Mean ± SEM. ns no significance. Two-way ANOVA). **E** Volcano plot showing differentially expressed genes in BxPC-3 cells following JOSD1 knockdown (upregulated, red; downregulated, blue). **F** Volcano plot showing differentially expressed genes in BxPC-3 cells following SUFU knockdown (upregulated, red; downregulated, blue). **G** Venn diagram showing overlapping genes between JOSD1 and SUFU knockdown in BxPC-3 cells. **H** Gene Ontology (GO) analysis of overlapping genes. **I** Kyoto Encyclopedia of Genes and Genomes (KEGG) pathway analysis of overlapping genes. **J** Heatmap of overlapping genes. **K** Validation of proliferation- and apoptosis-related genes among overlapping genes by RT-qPCR (*n* = 4. Mean ± SEM. *P < 0.05, ***P < 0.001, ****P < 0.0001. ns no significance. Two-way ANOVA).

To dissect the mechanism by which the JOSD1-SUFU axis regulates pancreatic cancer cell behavior, we first tested whether SUFU or JOSD1 depletion affects Hh signaling, given the canonical role of SUFU in this pathway. We therefore examined the expression of key transcription factors, GLI1 and GLI2, at both the mRNA and protein levels, as well as the mRNA level of the downstream target gene PTCH1, following SUFU or JOSD1 knockdown. GLI1 expression showed an upward trend at both the mRNA and protein levels but did not reach statistical significance, whereas GLI2 expression was significantly downregulated. In contrast, PTCH1 mRNA levels did not show a statistically significant change (Fig. 4A). To further assess whether SUFU-dependent cellular effects are mediated through GLI1, we evaluated the impact of GLI1 on pancreatic cancer cell growth. Given the low endogenous expression of GLI1 in BxPC-3 cells, we ectopically overexpressed GLI1, which was confirmed at both the mRNA and protein levels (Fig. 4B). GLI1 overexpression did not affect cell proliferation, as assessed by direct cell counting and CCK-8 assays (Fig. 4C, D). Taken together, these findings indicate that SUFU regulates pancreatic cancer cell growth through a non-canonical, Hh-independent mechanism.

Therefore, we sought to further explore the downstream molecular mechanisms by which the JOSD1-SUFU axis regulates pancreatic cancer cell behavior. To this end, we performed RNA-seq analysis following siRNA-mediated knockdown of endogenous JOSD1 or SUFU in BxPC-3 cells, with three biological replicates per condition. In the *JOSD1* knockdown group, we identified 888 differentially expressed genes (DEGs), comprising 584 upregulated and 304 downregulated genes (Fig. 4E). In the *SUFU* knockdown group, 705 DEGs were detected, with 389 upregulated and 316 downregulated genes (Fig. 4F). Overlap analysis revealed 245 DEGs shared between the two conditions (Fig. 4G). Functional enrichment analysis of these shared DEGs revealed significant enrichment in inflammation- and immune-related pathways, as well as biological processes associated with the negative regulation of cell proliferation and the positive regulation of apoptosis. These transcriptomic patterns were corroborated by coordinated regulation of proliferation/apoptosis-related genes in both knockdown conditions, as validated by RT-qPCR (Fig. 4H–K). These findings suggest that JOSD1 and SUFU constrain pancreatic cancer cell growth by orchestrating transcriptional programs governing cell proliferation and apoptosis.

### JOSD1 drives pancreatic cancer proliferation and tumorigenicity through SUFU

**Fig. 5:**
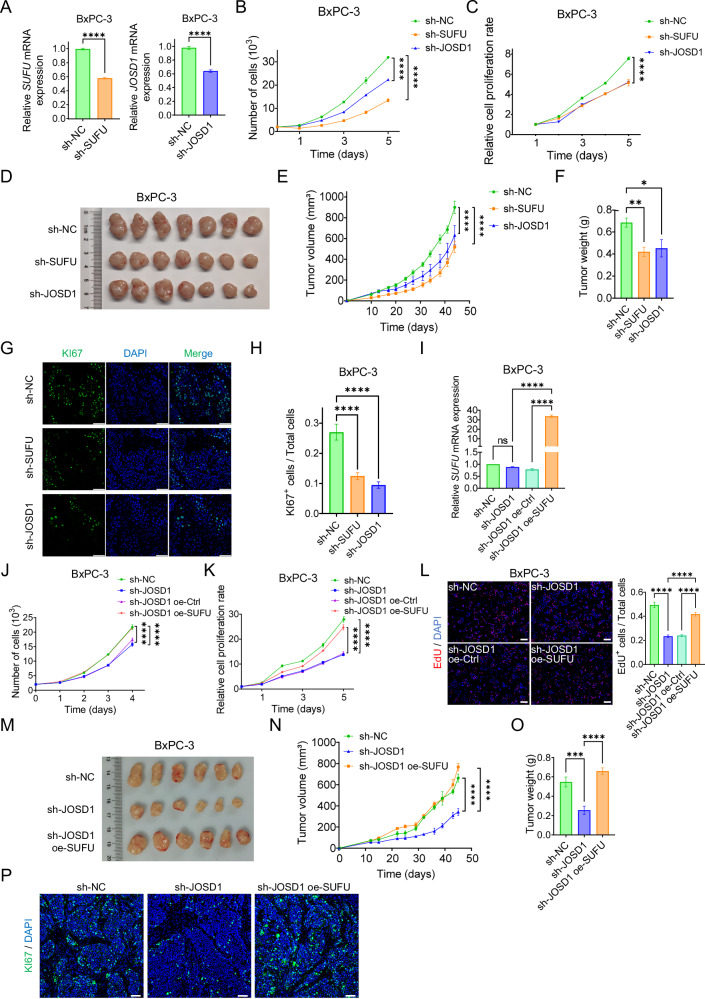
JOSD1 regulates tumorigenicity of pancreatic cancer cells through SUFU. **A** Knockdown efficiency of SUFU and JOSD1 in BxPC-3 cells following viral transduction was assessed by RT-qPCR (*n* = 4. Mean ± SEM. ****P < 0.0001. Unpaired Student’s *t* test). **B** Cell proliferation of BxPC-3 cells following SUFU and JOSD1 knockdown was assessed by cell counting (*n* = 3. Mean ± SEM. ****P < 0.0001. Two-way ANOVA). **C** Relative cell proliferation of BxPC-3 cells following SUFU and JOSD1 knockdown was assessed by CCK-8 assay (*n* = 6. Mean ± SEM. ****P < 0.0001. Two-way ANOVA). **D** Representative images of tumors formed by subcutaneous xenograft. **E** Statistics of tumor volume (*n* = 7. Mean ± SEM. ****P < 0.0001. Two-way ANOVA). **F** Statistics of tumor weight (*n* = 7. Mean ± SEM. *P < 0.05, **P < 0.01. One-way ANOVA). **G**, **H** KI67 staining showing proliferating (KI67-positive) cells in tumor tissues (bar, 50 µm. *n* = 16. Mean ± SEM. ****P < 0.0001. One-way ANOVA). **I** Overexpression efficiency of SUFU in JOSD1-knockdown BxPC-3 stable cells following viral transduction was assessed by RT-qPCR (*n* = 3. Mean ± SEM. ****P < 0.0001. One-way ANOVA). **J** Cell proliferation of BxPC-3 cells following SUFU rescue was assessed by cell counting (*n* = 3. Mean ± SEM. ****P < 0.0001. Two-way ANOVA). **K** Relative cell proliferation of BxPC-3 cells following SUFU rescue was assessed by CCK-8 assay (*n* = 5. Mean ± SEM. ****P < 0.0001. Two-way ANOVA). **L** EdU staining showing proliferating BxPC-3 cells following SUFU rescue (bar, 50 µm. *n* = 6. Mean ± SEM. ****P < 0.0001. One-way ANOVA). **M** Representative images of tumors formed by subcutaneous xenograft. **N** Statistics of tumor volume (*n* = 6. Mean ± SEM. ****P < 0.0001. Two-way ANOVA). **O** Statistics of tumor weight (*n* = 6. Mean ± SEM. ***P < 0.001, ****P < 0.0001. One-way ANOVA). **P** KI67 staining showing proliferating (KI67-positive) cells in tumor tissues (bar, 50 µm).

To determine whether JOSD1 and SUFU regulate pancreatic cancer tumorigenicity in vivo, we generated stable BxPC-3 cell lines with lentiviral-mediated knockdown of JOSD1 or SUFU. RT-qPCR analysis confirmed successful knockdown, showing significantly reduced *SUFU* and *JOSD1* mRNA levels in the respective cell lines (Fig. 5A). Consistent with prior siRNA-mediated results (Figs. 1F, H and 3E, G), both cell counting and CCK-8 assays demonstrated markedly impaired proliferation in *JOSD1* and *SUFU* knockdown stable cell lines (Fig. 5B, C). To evaluate tumorigenic potential, 2 × 10^6^ cells from each stable line were subcutaneously injected into nude mice, and tumor growth was monitored twice weekly. After 44 days, tumor samples were collected. Compared to the controls, both tumor volume and weight were significantly reduced in the *JOSD1* and *SUFU* knockdown groups (Fig. 5D–F). Consistently, immunohistochemical staining for KI67 revealed a significant reduction in proliferative cells in tumor tissues derived from both knockdown groups (Fig. 5G, H). These data demonstrate that JOSD1 and SUFU are required for pancreatic cancer cell proliferation and tumor growth in vivo.

To further determine whether SUFU mediates the effects of JOSD1, we performed rescue experiments by re-expressing SUFU in JOSD1-depleted cells. RT-qPCR confirmed successful restoration of SUFU expression following JOSD1 knockdown (Fig. 5I). Functional assays showed that SUFU re-expression partially rescued the proliferation defects induced by JOSD1 depletion, as evidenced by cell counting and CCK-8 assays (Fig. 5J, K). Consistently, EdU incorporation assays demonstrated a partial restoration of proliferative capacity upon SUFU re-expression (Fig. 5L). In vivo, SUFU re-expression markedly restored tumor growth in the subcutaneous xenograft model compared with JOSD1 knockdown alone (Fig. 5M–O). Concordantly, KI67 staining showed an increased proportion of proliferating cells in tumors with SUFU re-expression (Fig. 5P). Collectively, these results indicate that the tumor-suppressive effect of JOSD1 depletion is mediated through SUFU.

## Discussion

Pancreatic cancer is notably resistant to conventional therapies such as chemotherapy and radiotherapy [35, 36]. which severely limits treatment efficacy and contributes to its dismal prognosis. These challenges underscore the urgent need to explore the underlying biological characteristics of pancreatic cancer, including its genetic, epigenetic, and microenvironmental factors. In particular, identifying key regulatory genes and signaling pathways involved in tumor progression is essential for understanding its aggressive nature. Moreover, the development of targeted therapeutic strategies aimed at modulating these molecular drivers [35]. especially those that regulate cell proliferation, migration, and invasion, holds great promise for improving treatment outcomes. In this study, we define a functional relationship between JOSD1 and SUFU in pancreatic cancer. JOSD1 directly interacts with SUFU and stabilizes it by inhibiting its ubiquitination and subsequent degradation. Consistent with this, depletion of either JOSD1 or SUFU suppresses cell proliferation and migration while promoting apoptosis, whereas restoration of SUFU partially rescues the effects of JOSD1 loss, supporting a functional link between the two proteins. Together, these findings suggest that JOSD1 promotes pancreatic cancer progression through SUFU, and highlight this regulatory mechanism as a potential therapeutic target.

SUFU is a key negative regulator of the Hh signaling pathway, controlling cell proliferation, differentiation, and apoptosis by inhibiting the activity of downstream GLI transcription factor [37, 38]. It has been widely characterized as a tumor suppressor in multiple cancer types [39]. However, accumulating evidence suggests that SUFU can also exert pro-tumorigenic effects. For example, increased *Sufu* gene dosage accelerates cerebellar development and promotes medulloblastoma tumorigenesis [40]. In addition, SUFU has been reported to modulate GLI activity independently of upstream Hh signaling, thereby promoting pancreatic cancer cell growth [14]. Consistent with these observations, our findings support a pro-tumorigenic role of SUFU in pancreatic cancer. Notably, while these effects may still involve modulation of GLI activity, emerging evidence further indicates that SUFU can also participate in tumor progression through mechanisms independent of Hh signaling. For example, SUFU has been shown to regulate tumor cell ferroptosis sensitivity [13]. and migration [15]. independently of Hh signaling. In the present study, we extend these findings by showing that SUFU regulates pancreatic cancer cell growth through a non-canonical, Hh-independent mechanism. Together, these data position SUFU as a multifaceted regulator in cancer, with functions extending beyond its classical role in Hh signaling.

The regulation of SUFU is multifaceted and operates at multiple levels, reflecting its central role in cellular signaling and tumor progression. At the post-translational level, SUFU is extensively regulated by modifications that influence its stability, localization, and activity. Among these, ubiquitination-mediated degradation represents a major mechanism controlling SUFU protein turnover [16]. Multiple E3 ubiquitin ligases, including SCF (Fbxl17) [41]. SPOP [42]. and the Itch/β-arrestin2 complex [39]. have been shown to promote SUFU ubiquitination and proteasomal degradation. The activity and specificity of these ligases are further shaped by tumor type, microenvironmental cues, and SUFU mutational status. Beyond ubiquitination, SUFU function is also modulated by protein-protein interactions, which determine its ability to restrain GLI transcription factors or engage in non-canonical signaling pathways [13, 15, 43, 44]. However, how SUFU ubiquitination is reversed remains unclear. Here, we identify JOSD1 as a deubiquitinase that directly interacts with and stabilizes SUFU by preventing its ubiquitination-dependent degradation. This finding reveals a previously underappreciated layer of SUFU regulation and suggests that a dynamic balance between ubiquitination and deubiquitination governs SUFU protein stability and function in cancer.

Given that SUFU may regulate cellular functions through non-canonical, Hh-independent mechanisms, its downstream regulatory programs were explored using transcriptomic analysis. Alterations in SUFU or JOSD1 levels are associated with changes in gene expression programs related to cell proliferation and apoptosis. Beyond these processes, transcriptomic analyses also suggest a link between SUFU and inflammatory signaling, as genes upregulated upon SUFU or JOSD1 depletion are enriched in inflammation-related pathways. Inflammatory responses are closely linked to tumor progression, influencing cell survival, immune evasion, and microenvironment remodeling. JOSD1 has been implicated in immune regulation by negatively regulating type I interferon responses through deubiquitination and stabilization of SOCS1, supporting a broader role in inflammatory signaling [32]. Notably, SUFU has also been reported to regulate inflammation, for example, via modulation of TRAF6 phase separation [45]. suggesting that JOSD1 and SUFU may converge on the control of inflammatory signaling. Together, these observations suggest that stabilization of SUFU may contribute to the integration of multiple downstream signaling pathways, including proliferation, apoptosis, and inflammation, thereby shaping pancreatic cancer cell behavior. Further studies are required to define the mechanistic links between SUFU and inflammatory signaling and to determine the functional significance of these changes in tumor progression.

In conclusion, our study identifies SUFU as a critical regulator of pancreatic cancer cell behavior and reveals a previously unrecognized mechanism by which its stability is controlled. We demonstrate that JOSD1 directly interacts with and stabilizes SUFU by inhibiting its ubiquitination-dependent degradation, thereby sustaining its functional activity. Notably, SUFU exerts its effects through a non-canonical, Hedgehog-independent mechanism, influencing key cellular processes including proliferation, apoptosis, and tumor growth. Functional and rescue experiments further establish SUFU as a key downstream effector of JOSD1 in pancreatic cancer. Our study links SUFU function to ubiquitin-mediated proteostasis and highlights the JOSD1-SUFU pathway as a potential therapeutic target in pancreatic cancer.

## Data Availability

The data supporting the findings of this study are included in the article and supplementary files. The data for all bioinformatics analyses in this article were derived from public databases, including TCGA and GEPIA. RNA-seq data have been deposited in Sequence Read Archive of NCBI (accession code PRJNA1240927).
